# Reveal the Antimigraine Mechanism of Chuanxiong Rhizoma and Cyperi Rhizoma Based on the Integrated Analysis of Metabolomics and Network Pharmacology

**DOI:** 10.3389/fphar.2022.805984

**Published:** 2022-03-24

**Authors:** Zhiyao Zhu, Sha Wu, Yuxuan Wang, Jiayi Wang, Yujia Zhang

**Affiliations:** ^1^ School of Traditional Chinese Medicine, Capital Medical University, Beijing, China; ^2^ Beijing Key Lab of Traditional Chinese Medicine Collateral Disease Theory Research, Capital Medical University, Beijing, China

**Keywords:** network pharmacology, metabolomics, migraine, mechanism, *Cyperus* rotundus l, *Conioselinum anthriscoides* Chuanxiong

## Abstract

Migraine is a common neurological disorder that manifests as recurrent attacks of unilateral and throbbing headache. *Conioselinum anthriscoides* “Chuanxiong” (Apiaceae; Chuanxiong rhizoma) and *Cyperus* rotundus L. (Cyperaceae; Cyperi rhizoma) (CRCR), is a classic prescription for treating migraine. This study aimed to reveal the potential mechanisms of CRCR extract against migraine using integrated analysis of metabolomics and network pharmacology. Behavioral changes in the nitroglycerin rat migraine model were determined from von Frey withdrawal response. Untargeted serum metabolomics was used to identify the differentially expressed metabolites and metabolic pathways. The differentially expressed metabolites were analyzed to obtain the corresponding targets by a compound–reaction–enzyme–gene network. Network pharmacology was used to construct a compound–target–pathway network. The common targets of metabolomics and network pharmacology were further analyzed. Metabolomics analysis identified 96 differentially expressed metabolites and 77 corresponding targets. Network pharmacology analysis identified 201 potential targets for CRCR against migraine. By intersecting 77 targets with 201 targets, monoamine oxidase A (MAO-A), monoamine oxidase B (MAO-B), and catechol-*O*-methyltransferase (COMT) were identified as the common targets, and MAO-A, MAO-B, and COMT were involved in the tyrosine metabolism pathway. Further experiments demonstrated that the contents of MAO-A and COMT were significantly increased in serum and brainstem tissue of the migraine rats. CRCR extract significantly decreased the contents of MAO-A and COMT, while no significant difference was found in MAO-B. Metabolomics analysis indicated that the contents of 3,4-dihydroxyphenylacetate (DOPAC) and 3-(4-hydroxyphenyl)pyruvate (HPP) were significantly increased in the migraine rats, and CRCR extract caused significant decreases in DOPAC and HPP. Interestingly, DOPAC and HPP were two differentially expressed metabolites involved in the tyrosine metabolism pathway. Correlation analysis showed that DOPAC and HPP were highly positively correlated with MAO-A and COMT. Taken together, two key differentially expressed metabolites (DOPAC and HPP), two key targets (MAO-A and COMT), and one relevant metabolic pathway (tyrosine metabolism) showed great importance in the treatment of migraine. This research could provide a new understanding of the potential mechanism of CRCR against migraine. More attentions should be paid into the tyrosine metabolism pathway in future studies.

## Introduction

Migraine is a chronic paroxysmal neurological disorder characterized by multiphase attacks of head pain and a myriad of neurological symptoms (Charles, 2018). The sensitization of the trigeminovascular system followed by dural neurogenic inflammation plays a crucial role in the pathogenesis of migraine (Dodick, 2018; Ashina, 2020). Cerebral energy deficiency, metabolic abnormality, and oxidative stress are also involved in migraine attacks (Gross et al., 2019; Haanes and Edvinsson, 2019). Migraine mechanisms remain to be fully uncovered for various reasons; it still needs to be sharply focused.

Botanical drugs have been widely used in the treatment of migraine attacks (Hou et al., 2017; Lopresti et al., 2020). *Conioselinum anthriscoides* “Chuanxiong” (Apiaceae; Chuanxiong rhizoma) (known as Chuanxiong rhizoma in Chinese) is used to treat migraine and cerebral ischemic stroke. *Cyperus* rotundus L. (Cyperaceae; Cyperi rhizoma) (known as Cyperi rhizoma in Chinese) is usually used to treat depression, epilepsy, and dysmenorrhea. Chuanxiong rhizoma and Cyperi rhizoma (abbreviated as CRCR), in a weight ratio of 1:2, is a classic prescription for treating migraine dating back to the Danxi Xinfa treatise in the Yuan Dynasty (AD 1347) of China. CRCR has the traditional clinical efficacy in activating blood circulation, dispelling wind, and relieving pain. Modern studies discovered analgesic, anti-inflammatory, antioxidant, anticerebral ischemia, and neuroprotective activities in CRCR (Peerzada et al., 2015; Chen et al., 2018). In our previous study, CRCR can alleviate allodynia in the migraine rats by regulating the releasing of vasoactive substances, neurotransmitters, and neuropeptides, consequently relieving neurogenic inflammation (Wu et al., 2019; Guo et al., 2020). However, the underlying molecular mechanism of CRCR against migraine has yet to be well understood, which impedes its clinical application.

Metabolomics is a thriving approach to obtain an integral metabolome in biological systems, which has made great breakthroughs in disease prevention, control, and diagnosis (Dong et al., 2020; Li et al., 2020). There is growing interests in the network pharmacology, which has an advantage in elucidating the potential molecular mechanisms based on a drug–disease–target network (Li et al., 2019). Recently, the integrated analysis of metabolomics and network pharmacology has been applied to elucidate the protein–metabolite interactions, which brings great inspiration to the mechanism research (Wei et al., 2020; Yang et al., 2020; Zhang et al., 2020).

In this work, an integrated strategy of metabolomics and network pharmacology was used to reveal the antimigraine mechanism of CRCR. First, untargeted serum metabolomics was used to identify the differentially expressed metabolites and metabolic pathways based on UHPLC-Q-extractive MS analysis. Second, the identified differentially expressed metabolites were analyzed to obtain the corresponding targets by a compound–reaction–enzyme–gene network. Third, network pharmacology was used to construct a compound–target–pathway network and obtain potential targets for CRCR against migraine. Finally, the common targets of metabolomics and network pharmacology were further analyzed. Collectively, this study will hopefully contribute to a better understanding of the potential mechanism of CRCR against migraine.

## Materials and Methods

### Materials and Chemicals

Reference standards of ferulic acid (CAS 1135-24-6), 3-n-butylphthalide (CAS 6066-49-5), senkyunolide A (CAS 63038-10-8), Z-ligustilide (CAS 81944-09-4), Z-3-butylidenephthalide (CAS 72917-31-8), cyperotundone (CAS 3466-15-7), nookatone (CAS 4674-50-4), and α-cyperone (CAS 473-08-5) were purchased from PUSH Bio-technology (Chengdu, Sichuan, China). All these chemicals were with a purity of greater than 98%. Nitroglycerin injections (NTGs) were obtained from Beijing Yimin Pharmaceutical Co., Ltd. (Beijing, China). Sumatriptan succinate tablets were purchased from Tianjin Huajin Pharmaceutical Co., Ltd. (Tianjin, China). Rat 5-HT, CGRP, NO, and NOS ELISA kits were purchased from Nanjing Jiancheng Bioengineering Institute (Nanjing, Jiangsu, China). Rat MAO-A, MAO-B, and COMT ELISA kits were purchased from Shanghai Enzyme-Linked Biotechnology Co., Ltd. (Shanghai, China). HPLC-grade methanol and acetonitrile were obtained from Fisher Scientific Inc. (Fairlawn, NJ, United States). Ultrapure water was produced by a Milli-Q Reagent Water System (Bedford, MA, United States).

### Preparation and Quality Control of *Chuanxiong rhizoma* and *Cyperi rhizoma* extract

Botanical drugs of Chuanxiong rhizoma (Batch No. 2001021) and Cyperi rhizoma (Batch No. D1908009) were obtained from Sichuan Neautus Traditional Chinese Co., Ltd. (Chengdu, Sichuan, China). They were identified by Dr. Li Ma (Capital Medical University, Beijing, China) according to the 2020 edition of Chinese Pharmacopoeia. Voucher specimens were deposited in the Beijing Key Lab of Traditional Chinese Medicine Collateral Disease Theory Research of Capital Medical University.

Chuanxiong rhizoma (62.5 g) and 125.0 g of Cyperi rhizoma were powdered, and then extracted by heating reflux with six volumes of 70% ethanol for 1 h and repeated twice. After filtered, the extraction solution was concentrated at 50°C using a vacuum rotary evaporator (N1100, EYELA, Tokyo, Japan) and then dried in vacuum for the collection of dry extract. The yield of the dry extract was 20.71%.

Eight main components were selected to be accurately quantified in the CRCR extract by HPLC according to our previous method (Guo et al., 2018). The contents of ferulic acid, senkyunolide A, 3-n-butylphthalide, Z-ligustilide, Z-3-butylidenephthalide, cyperotundone, nookatone, and α-cyperone were 0.88, 6.33, 3.37, 4.64, 0.96, 13.70, 0.16, and 0.09 mg/g in the CRCR extract, respectively.

### Animal Experiment

Male Sprague–Dawley rats (200 ± 20 g) were purchased from Beijing Vital River Laboratory Animal Technology Co., Ltd. (Beijing, China). Rats were housed in a temperature- and humidity-controlled environment. Rats were divided into four groups: control, NTG, sumatriptan, and CRCR groups. Rats were treated orally with saline at a dose of 10 ml/kg in the control and NTG groups, sumatriptan succinate tablets at a dose of 5.83 mg/kg in the sumatriptan group, and CRCR extract at a dose of 6.6 g/kg in the CRCR group for five consecutive days. After the last oral administration, rats were subcutaneously injected with 10 mg/kg of NTG to induce migraine attack. Two hours after NTG injection, the mechanical sensitivity thresholds were assessed by von Frey tests. Subsequently, rats were anesthetized by isoflurane inhalation. Blood was collected from the abdominal aorta and centrifuged at 4°C at 3,000 rpm for 15 min to obtain serum samples. Rats were sacrificed by cervical dislocation, and brainstem was separated on ice, and stored at −80°C until further analysis.

### Measurement of Mechanical Thresholds

Cutaneous allodynia is a typical symptom of migraine, with a crucial role in directing optimal treatment for migraine attacks (Landy et al., 2004). Cutaneous allodynia was measured by von Frey monofilaments by assessing the mechanical thresholds in the periorbital region of a rat. In brief, rats were placed in a plastic box to acclimate the situation before testing. Facial allodynia on the periorbital region of a rat was assessed by Electronic Von Frey Filaments (BIO-EVF3, BIOSEB, Vitrolles, France). The force values ranged from 0 to 500 g. A positive response was defined as a sharp retraction of the head or scratching the face with the forepaw (Wang S. et al., 2018). The test was repeated three times with an interval of at least 20 s, and the average value was calculated as the final result.

### Sample Preparation for Metabolomics Study

An aliquot of 100 µl of thawed serum sample was deproteinized with 400 µl of MeOH:ACN (1:1, *v/v*). After vortex mixing for 30 s and sonication for 30 min at 5°C, a sample was kept at −20°C for 30 min to improve protein precipitation. The sample was centrifuged at 13,000 ×*g* for 15 min at 4°C. The supernatant was evaporated at 37°C under a gentle nitrogen stream and then redissolved in 100 µl of ACN:H_2_O (1:1, *v/v*). After vortex mixing for 30 s and sonication for 5 min at 5°C, the sample was centrifuged at 13,000 ×*g* for 10 min at 4°C, and 2 µl of supernatant was subjected to UHPLC-Q-extractive MS analysis. For method validation, a pooled quality control (QC) sample was prepared by mixing an equal volume (20 µl) of each serum sample in parallel as above and inserted at regular intervals into the sequence.

### UHPLC-Q-Extractive MS Analysis

Metabolomics analysis was performed by using a UHPLC system coupled with a Q Exactive hybrid quadrupole Orbitrap mass spectrometer (Thermo Fisher Scientific, CA, United States). Chromatographic separation was performed by using an ACQUITY UPLC HSS T3 column (2.1 × 100 mm, 1.8 µm, Waters, Milford, CT, United States) at 40°C. The mobile phase consisted of acetonitrile/water (5:95, *v/v*, solvent A) and acetonitrile/isopropanol/water (47.5:47.5:5, *v/v/v*, solvent B), both containing 0.1% formic acid. The flow rate was 0.4 ml/min. The elution gradient program was used as follows: 0–3.5 min, 100%–75.5% A; 3.5–5.0 min, 75.5–35% A; 5.0–5.5 min, 35%–0% A; 5.5–7.4 min, 0%–0% A; 7.4–7.6 min, 0%–48.5% A; 7.6–7.8 min, 48.5%–100% A; 7.8–10.0 min, 100%–100% A. The MS parameters were used as follows: spray voltage, ±3.5 KV; sheath gas flow rate, 50 arb; auxiliary gas flow rate, 13 arb; capillary temperature, 325°C; heater temperature, 425°C. The MS scan range was operated with the mass m/z 70–1,050 Da and with a 0.2-s accumulation time.

### Method Validation of UHPLC-Q-Extractive MS

**FIGURE 1:**
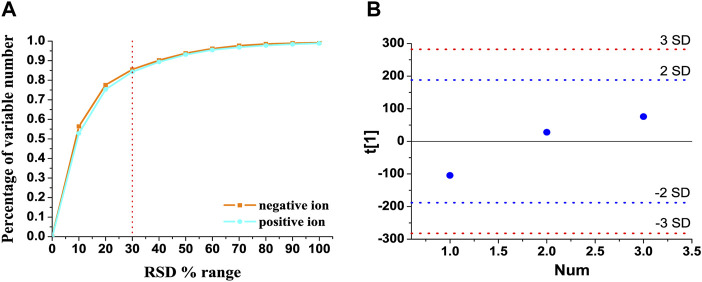
Method validation of UHPLC-Q-extractive MS analysis. **(A)** RSD distribution of accumulative percentage of detected variable numbers in the pooled quality control (QC) samples. **(B)** Time-series dependency of the first PCA component t[1] of QC samples (blue) within the whole analytical run. Blue and red lines indicate the 2 and 3 SD limits of peak area intensities, respectively.

The total ion chromatograms of QC samples in the positive and negative ion modes are presented in Supplementary Figures S1A,B, respectively. In order to reduce the false-positive rate caused by systematic errors, metabolic features detected in less than 80% of all the QC samples were removed, together with the features whose relative standard deviation of peak intensity was more than 30% in all the QC samples (Li et al., 2020). As shown in Figures 1A, 84.25% and 85.49% of the variables had an RSD% of less than 30% in the positive and negative ion modes, respectively. As shown in Figure 1B, three QC samples, which were inserted at every six samples, were within twice the standard deviation in the score plot. These results indicated that the established UHPLC-Q-extractive MS method was stable and reliable for metabolomics analysis.

### Data Processing and Analysis

The raw MS data were preprocessed for peak picking, peak alignment, and peak annotation. A peak list was generated, which contained mass-to-charge ratio (m/z), retention time, and peak intensity. Multivariate statistical analysis was used to identify the differentially expressed metabolites by SIMCA-P 13.0 software (Umetrics, Umea, Sweden). The differentially expressed metabolites were then identified based on the exact molecular weight and MS/MS spectrum based on online databases, e.g., Human Metabolome Database (HMDB, http://www.hmdb.ca), METLIN (http://metlin.scripps.edu) and MycompoundID (http://www.mycompoundid.org). Metabolite enrichment analysis and pathway analysis were performed by Metaboanalyst 5.0 (http://www.metaboanalyst.ca). The identified differential metabolites were used to construct a compound–reaction–enzyme–gene network in MetScape plugin in Cytoscape 3.7.1 software (Shannon et al., 2003).

### Network Pharmacology Analysis

Compounds were retrieved from the Traditional Chinese Medicine Systems Pharmacology Database (https://tcmsp-e.com/, TCMSP) and were manually replenished based on literature. Oral bioavailability (OB) and drug likeness (DL) were used to identify bioactive molecules. If a compound meets the criteria of OB ≥30% and DL ≥0.18, it will be chosen as a candidate molecule for further analysis. The SMILE structure of this molecule was imported into the Swiss Target Prediction database (http://www.swisstargetprediction.ch/) to obtain the corresponding targets. Furthermore, migraine-related gene targets were acquired from GeneCards (https://www.genecards.org/), Online Mendelian Inheritance in Man (OMIM, https://omim.org/), DisGeNet (https://www.disgenet.org/), Therapeutic Target Database (TTD, http://db.idrblab.net/ttd/), and Drugbank (https://go.drugbank.com/).

Migraine-related gene targets (2,992) were obtained from GeneCards database at first, and then 2,041 migraine-related gene targets were retained based on the median of the relevance score values. Migraine-related gene targets (48, 512, and 35) were obtained from OMIM, DisGeNet, and TTD databases, respectively. From Drugbank database, 244 migraine-related protein targets were acquired at first, and 185 corresponding gene targets were obtained using Uniprot database (https://www.uniprot.org/). Thus, a total of 1,801 migraine-related gene targets were retained by intersecting these targets from five different databases. The potential targets responsible for CRCR against migraine were acquired by intersecting CRCR-related gene targets with migraine-related gene targets. A protein–protein interaction network was constructed by STRING 11.0 (https://string-db.org/). The Gene Ontology (GO) and Kyoto Encyclopedia of Genes and Genomes (KEGG) enrichment analyses were performed by ClueGO plugin in Cytoscape 3.7.1 software.

### ELISA Analysis

The contents of 5-HT, CGRP, NO, NOS, MAO-A, MAO-B, and COMT in rat were analyzed with ELISA kits following the manufacturer’s instructions. Absorbance was measured in the SpectraMax iD3 Multi-Mode Microplate Readers (Molecular Devices, San Jose, CA, United States).

### Statistical Analysis

Data were expressed as mean ± SD. Statistical analysis was performed by one-way ANOVA followed by multiple comparisons using GraphPad Prism 7.0 software (GraphPad software Inc., La Jolla, CA, United States). A *p*-value of <0.05 was considered to be statistically significant. Pearson correlation analysis was performed using SPSS 17.0 software (SPSS Inc., Chicago, IL, United States).

## Results

### Effect of *Chuanxiong rhizoma* and *Cyperi rhizoma* on Migraine

**FIGURE 2:**
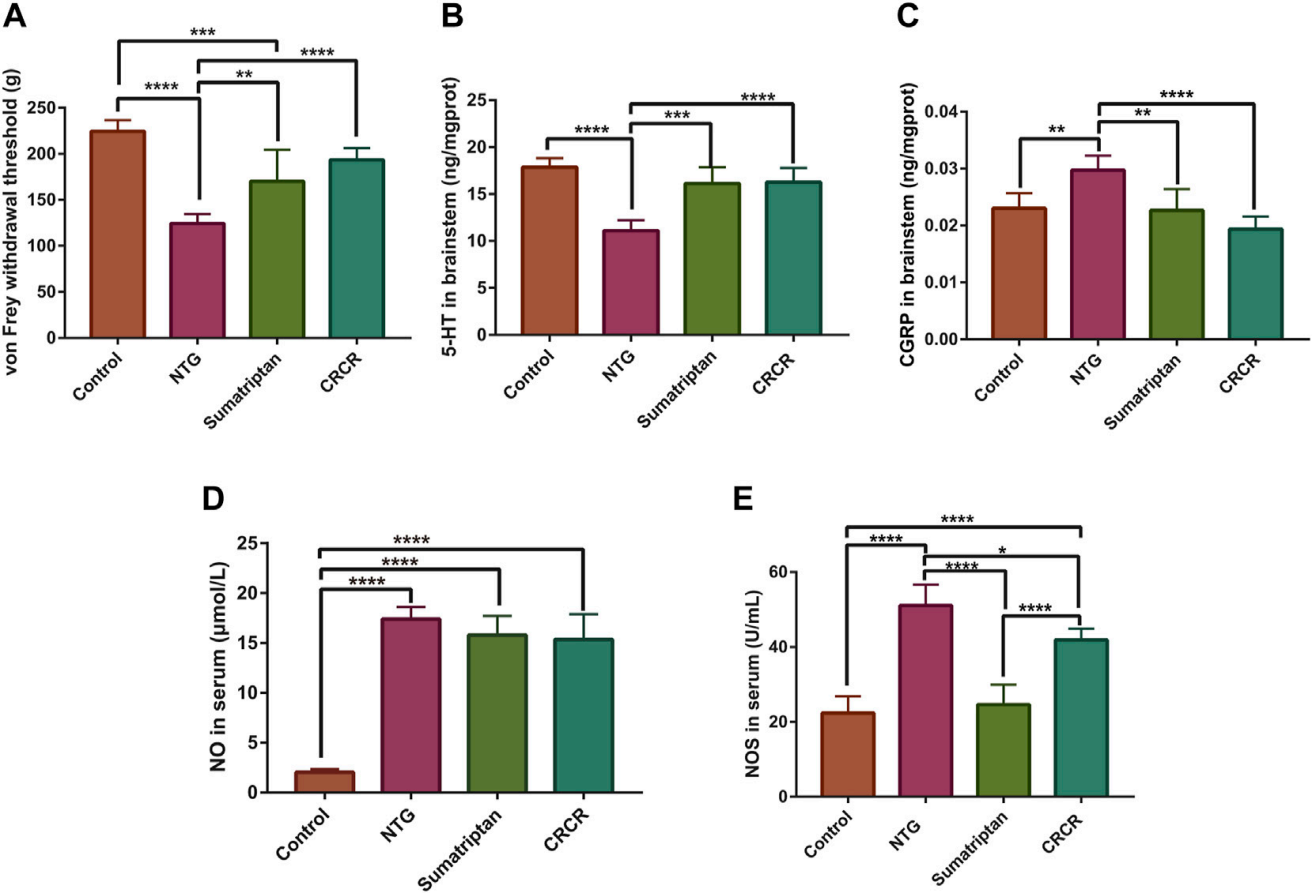
Measurement of mechanical thresholds and pharmacological indexes. **(A)** The thresholds of von Frey mechanical stimulation. **(B)** 5-Hydroxytryptamine (5-HT) in the rat brainstem. **(C)** Calcitonin gene-related peptide (CGRP) in the rat brainstem. **(D)** Nitric oxide (NO) in the rat serum. **(E)** Nitric oxide synthase (NOS) in the rat serum. Data are expressed as mean ± S.D. *p < 0.05, **p < 0.01, ***p < 0.001, ****p < 0.0001.

As shown in Figure 2A, the mechanical threshold was significantly decreased in the migraine rats (*p* < 0.0001), and CRCR significantly elevated the mechanical threshold (*p* < 0.0001). Figures 2B–E show the effect of CRCR on the pharmacological indexes. Significant reduction of serotonin [5-hydroxytryptamine (5-HT)] and significant improvement of calcitonin gene-related peptide (CGRP), nitric oxide (NO), and nitric oxide synthase (NOS) were observed in the migraine rats compared with the control rats (*p* < 0.0001, *p* < 0.01, *p* < 0.0001, *p* < 0.0001). With the treatment of CRCR, 5-HT was significantly increased (*p* < 0.0001), CGRP and NOS were significantly decreased (*p* < 0.0001, *p* < 0.05), which indicated CRCR had a good antimigraine effect on NTG-induced migraine rats.

### Metabolic Profiles of Serum Samples

**FIGURE 3:**
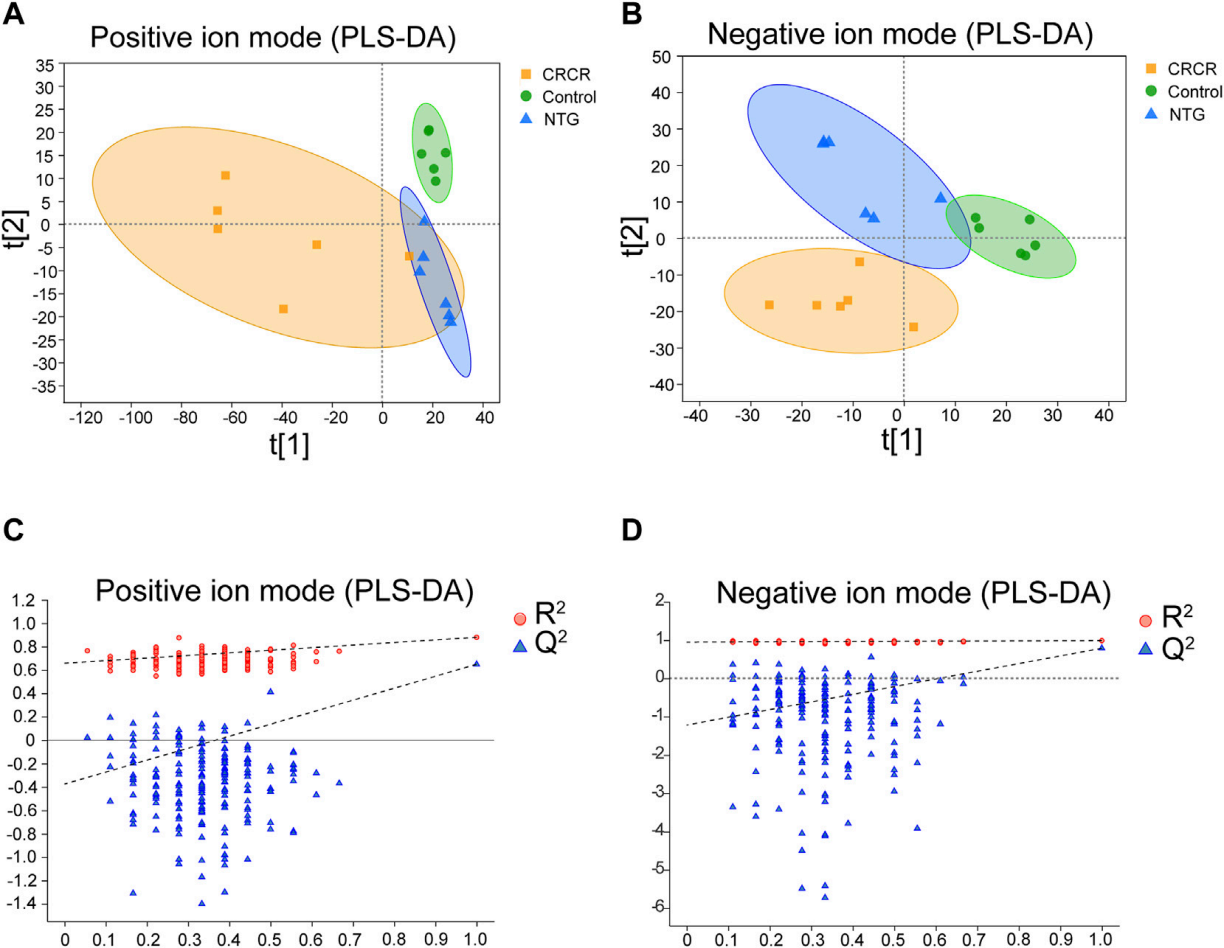
Partial least squares discriminate analysis (PLS-DA) score plots and the corresponding permutation test plots based on the UHPLC-Q-extractive MS data of control, nitroglycerin injection (NTG), and *Conioselinum anthriscoides* “Chuanxiong” (Apiaceae; Chuanxiong rhizoma) and *Cyperus* rotundus L. (Cyperaceae; Cyperi rhizoma) (CRCR) groups in the positive **(A,C)** and negative ion modes **(B,D)**.

The metabolic profiles of serum samples were acquired by the UHPLC-Q-extractive MS data. After *Pareto* scaling and mean-centering pretreatment, a partial least squares discriminate analysis (PLS-DA) model was applied to identify the clustering property. As shown in Figures 3A, B, PLS-DA score plots exhibited relatively tight clusters and clear discrimination among control, NTG, and CRCR groups in both positive and negative ion modes. Subsequently, permutation tests were utilized to validate the established PLS-DA model. As shown in Figures 3C, D, the model had good *R* ^2^ and Q^2^ values after 200 response permutation tests, indicating that the PLS-DA model was reliable with a low risk of overfitting.

### Identification of Differentially Expressed Metabolites and Metabolic Pathway

**FIGURE 4:**
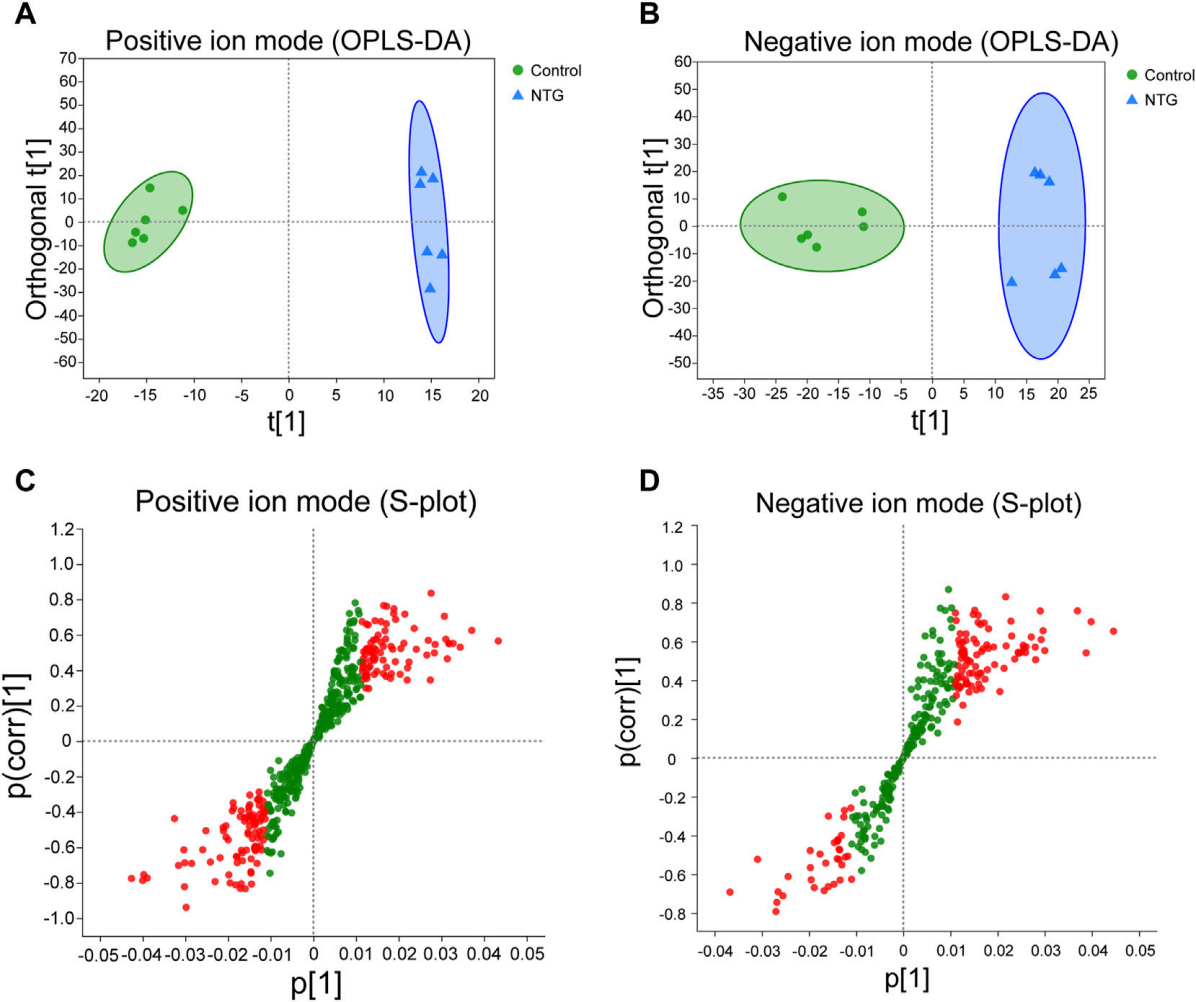
Orthogonal partial least squares discriminate analysis (OPLS-DA) score plots and the corresponding *S*-plots based on the UHPLC-Q-extractive MS data of control and NTG groups in the positive **(A,C)** and negative ion modes **(B,D)**. Red dot represents the differentially expressed metabolites with VIP > 1 and *p* < 0.05.

Orthogonal partial least squares discriminate analysis (OPLS-DA) was applied to analyze the overall metabolic distinction. A clear grouping trend between control and model samples could be observed in the positive ion mode (*R* ^*2*^ *X* = 0.487, *R* ^*2*^ *Y* = 0.992, *Q* ^*2*^ = 0.647, Figure 4A) and negative ion mode (*R* ^*2*^ *X* = 0.359, *R* ^*2*^ *Y* = 0.953, *Q* ^*2*^ = 0.697, Figure 4B) in the OPLS-DA score plots. The variable importance in the projection (VIP) value (VIP > 1) and statistical difference (*p* < 0.05) were combined as the critical indicators to identify differentially expressed metabolites. A total of 1,897 metabolites were identified between the control and NTG groups, including 901 metabolites in the positive ion mode (Figure 4C) and 996 metabolites in the negative ion mode (Figure 4D). Based on MS/MS fragments, retention behavior, and online databases, 96 differentially expressed metabolites (60 metabolites in the positive ion mode and 36 metabolites in the negative ion mode) were identified.

**FIGURE 5:**
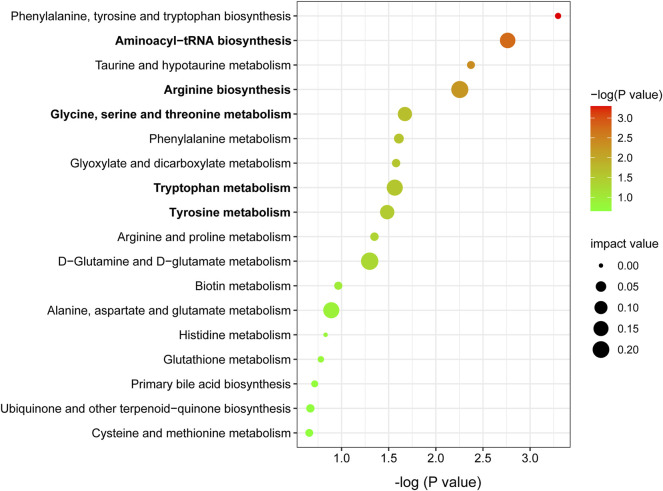
Metabolic pathway enrichment analysis of differentially expressed metabolites. Node size is based on impact value, node color is based on −log (P) value. The pathways marked in bold are statistically different with impact value >0.1 and *p* < 0.05.

Metabolic pathway analysis was carried out by importing 96 differentially expressed metabolites into the Metaboanalyst 5.0. By setting the threshold as impact value >0.1 and *p*-value <0.05, five differential metabolic pathways were found, including aminoacyl-tRNA biosynthesis, arginine biosynthesis, glycine, serine and threonine metabolism, tryptophan metabolism, and tyrosine metabolism (Figure 5). As shown in Table 1, seven differentially expressed metabolites were found to be enriched in the above metabolic pathways.

**TABLE 1:** Five key metabolic pathways and the corresponding differentially expressed metabolites in metabolomics.

No	Pathways	Total	Hits	Impact	*p*-Value	Metabolites
1	Aminoacyl-tRNA biosynthesis	52	3	0.162	0.002	L-glutamate, L-serine, L-tryptophan
2	Arginine biosynthesis	23	2	0.211	0.005	L-citrulline, L-glutamate
3	Glycine, serine, and threonine metabolism	47	2	0.127	0.021	L-serine, L-tryptophan
4	Tryptophan metabolism	54	2	0.179	0.027	L-tryptophan, indole-3-acetamide
5	Tyrosine metabolism	60	2	0.130	0.033	3,4-dihydroxyphenylacetate (DOPAC)
						3-(4-hydroxyphenyl)pyruvate (HPP)

### Network Pharmacology Analysis

**FIGURE 6:**
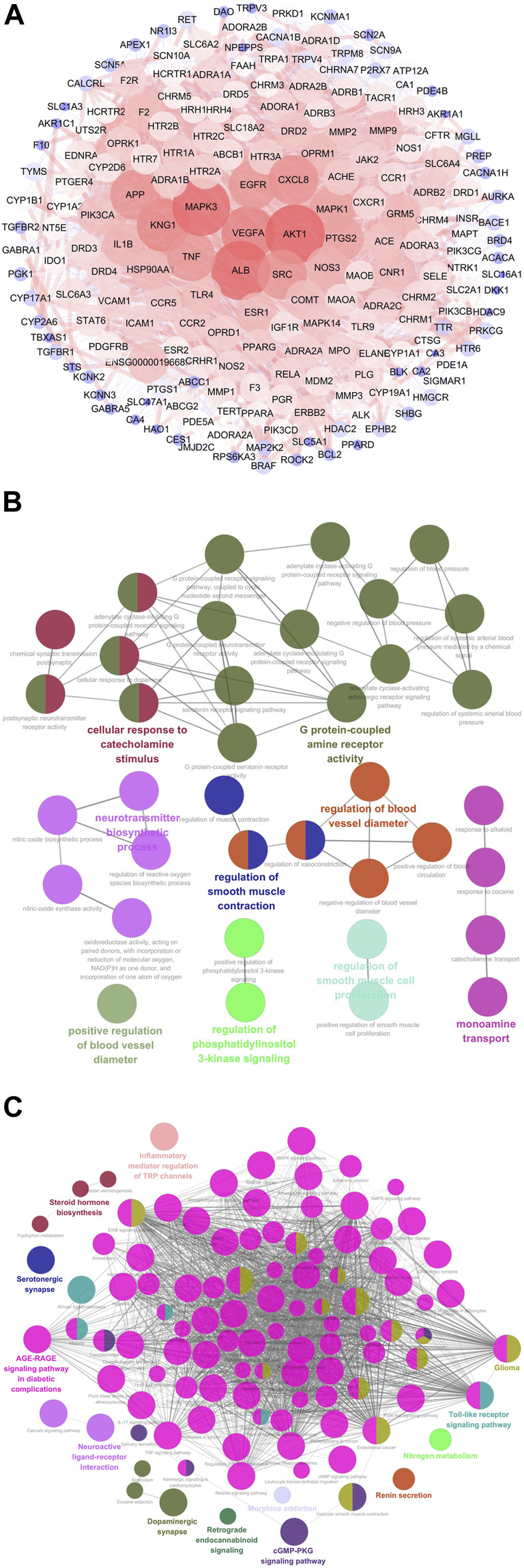
Network pharmacology analysis of CRCR in migraine treatment. **(A)** The protein–protein interaction network of 201 potential targets responsible for CRCR against migraine. The nodes represent targets, while the edges represent the association between nodes and nodes, and the node size represents degree value. **(B)** The Gene Ontology (GO) biological process analysis of potential targets by ClueGo. **(C)** The Kyoto Encyclopedia of Genes and Genomes (KEGG) enrichment analysis of potential targets by ClueGo.

A total of 31 compounds from CRCR were collected to obtain 605 target genes from the Swiss Target Prediction database. Migraine-related gene targets (1,801) were acquired from GeneCards, OMIM, DisGeNet, TTD, and Drugbank databases. After intersecting 605 targets with 1,801 targets, 201 targets were identified as the potential targets for CRCR against migraine. A protein–protein interaction network was constructed by the Cytoscape software. Figure 6A represents a whole view of 201 target genes. *AKT1*, *ALB*, *MAPK3*, *VEGFA*, *KNG1*, *SRC*, *CXCL8*, *APP*, *EGFR*, and *TNF* were the top 10 genes with high degree values. GO and KEGG enrichment analyses were performed by ClueGO. The top terms in GO biological process were G protein-coupled amine receptor activity, cellular response to catecholamine stimulus, neurotransmitter biosynthetic process, regulation of blood vessel diameter, monoamine transport, and so on (Figure 6B). The top terms in KEGG enrichment pathway were inflammatory mediator regulation of TRP channels (map04750), steroid hormone biosynthesis (map00140), serotonergic synapse (map04726), neuroactive ligand–receptor interaction (map04080), dopaminergic synapse (map04728), nitrogen metabolism (map00910), cGMP–PKG signaling pathway (map04022), Toll-like receptor signaling pathway (map04620), AGE–RAGE signaling pathway in diabetic complications (map04933), retrograde endocannabinoid signaling (map04723), glioma (map05214), and renin secretion (map04924) (Figure 6C). Functional annotation and pathway enrichment analysis indicated that multiple pathways were closely involved in the treatment of migraine by CRCR.

### Integrated Analysis of Metabolomics and Network Pharmacology

**FIGURE 7:**
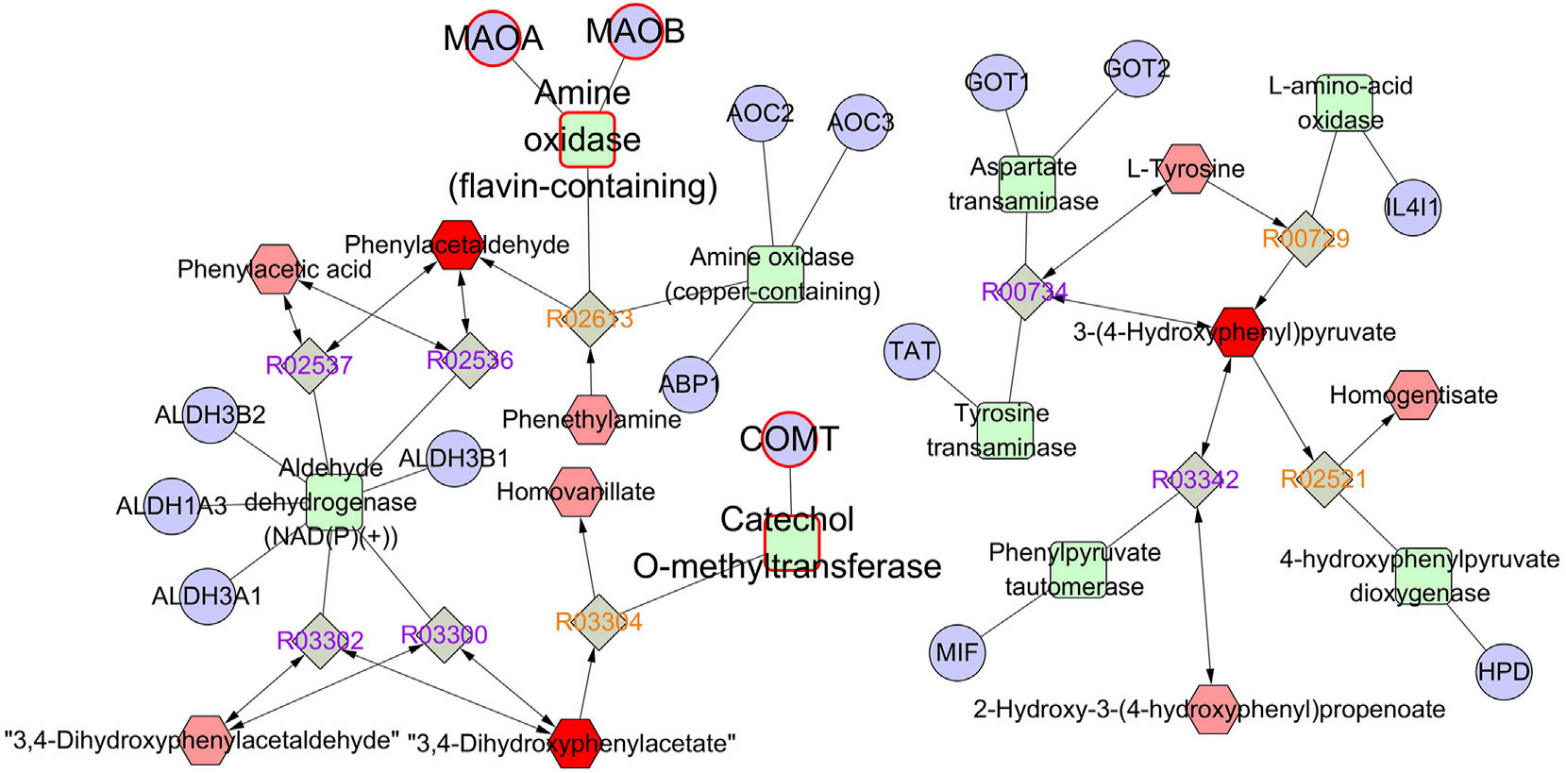
The compound–reaction–enzyme–gene network of tyrosine metabolism. The red hexagons, gray diamonds, green round rectangles, and purple circles represent active compounds, reactions, proteins, and genes, respectively.

The identified differentially expressed metabolites were imported into Metscape to obtain a compound–reaction–enzyme-–gene network, which visualized the interaction among metabolites, pathways, enzymes, and genes. As shown in Supplementary Table S1, 77 potential target genes were found in the compound–reaction–enzyme–gene network. By intersecting 77 targets in the compound–reaction–enzyme–gene network with 201 targets in the protein–protein interaction network, three key targets were identified, including monoamine oxidase A (MAO-A), monoamine oxidase B (MAO-B), and catechol-*O*-methyltransferase (COMT). As shown in Figure 7, MAO-A, MAO-B, and COMT were clearly involved in the tyrosine metabolism pathway. 3,4-Dihydroxyphenylacetate (DOPAC), 3-(4-hydroxyphenyl)pyruvate (HPP), and phenylacetaldehyde were the key differentially expressed metabolites in the tyrosine metabolism pathway.

### Analysis of Common Targets and Differentially Expressed Metabolites

**FIGURE 8:**
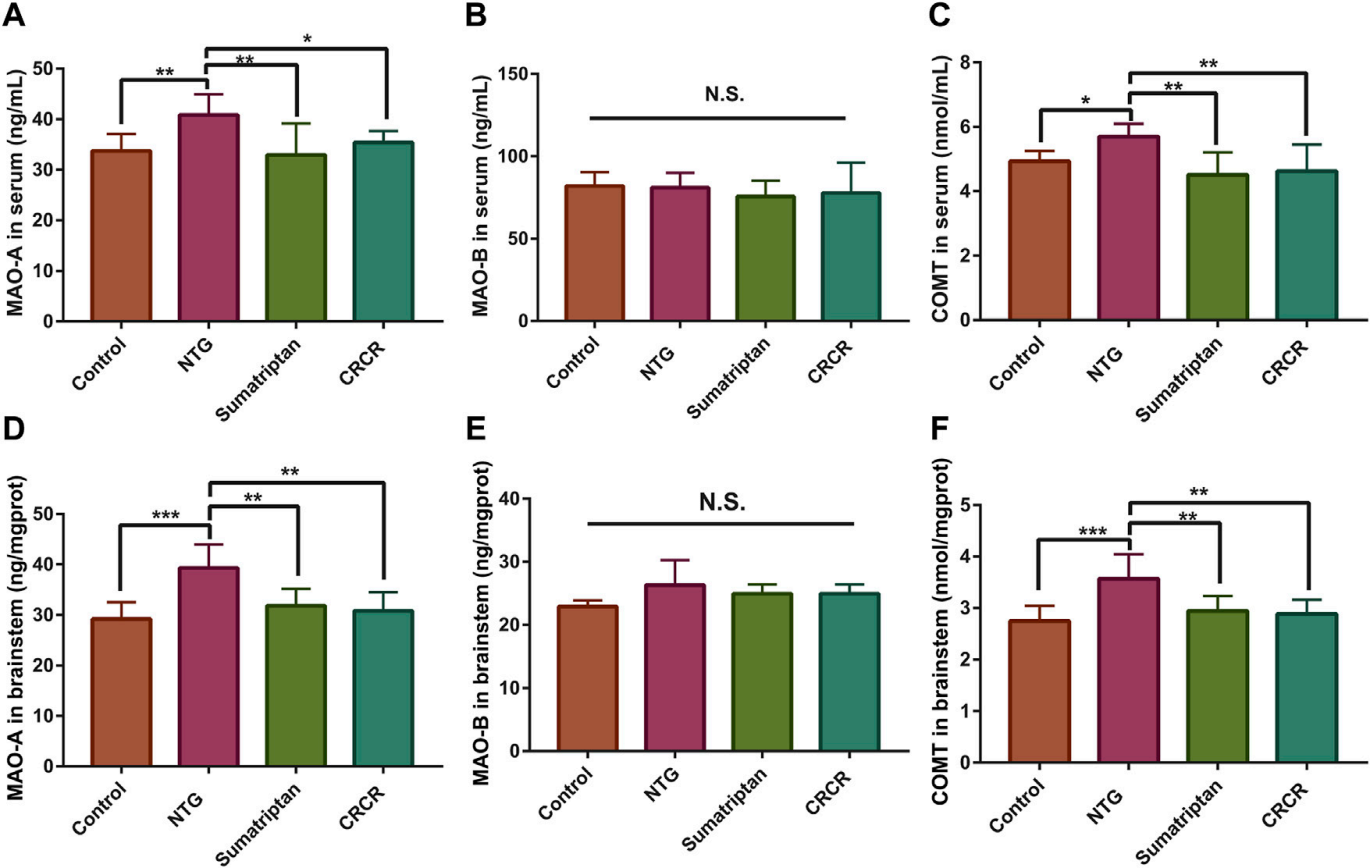
The contents of monoamine oxidase A (MAO-A), monoamine oxidase B (MAO-B), and catechol-*O*-methyltransferase (COMT) in rat serum and brainstem. **(A)** MAO-A in serum. **(B)** MAO-B in serum. **(C)** COMT in serum. **(D)** MAO-A in brainstem. **(E)** MAO-B in brainstem. **(F)** COMT in brainstem. Data are expressed as mean ± S.D. *p < 0.05, **p < 0.01, ***p < 0.001, ****p < 0.0001.

MAO-A, MAO-B, and COMT were identified as the common targets based on metabolomics and network pharmacology analysis. The contents of MAO-A, MAO-B, and COMT in rat serum and brainstem tissue were analyzed by ELISA. As shown in Figure 8, compared with the control rats, the contents of MAO-A and COMT were significantly increased in the migraine rats. Treatment with CRCR significantly decreased the contents of MAO-A and COMT. However, there was no significant difference in MAO-B. These results showed that CRCR extract significantly inhibited the upregulation of MAO-A and COMT in the migraine rats.

**FIGURE 9:**
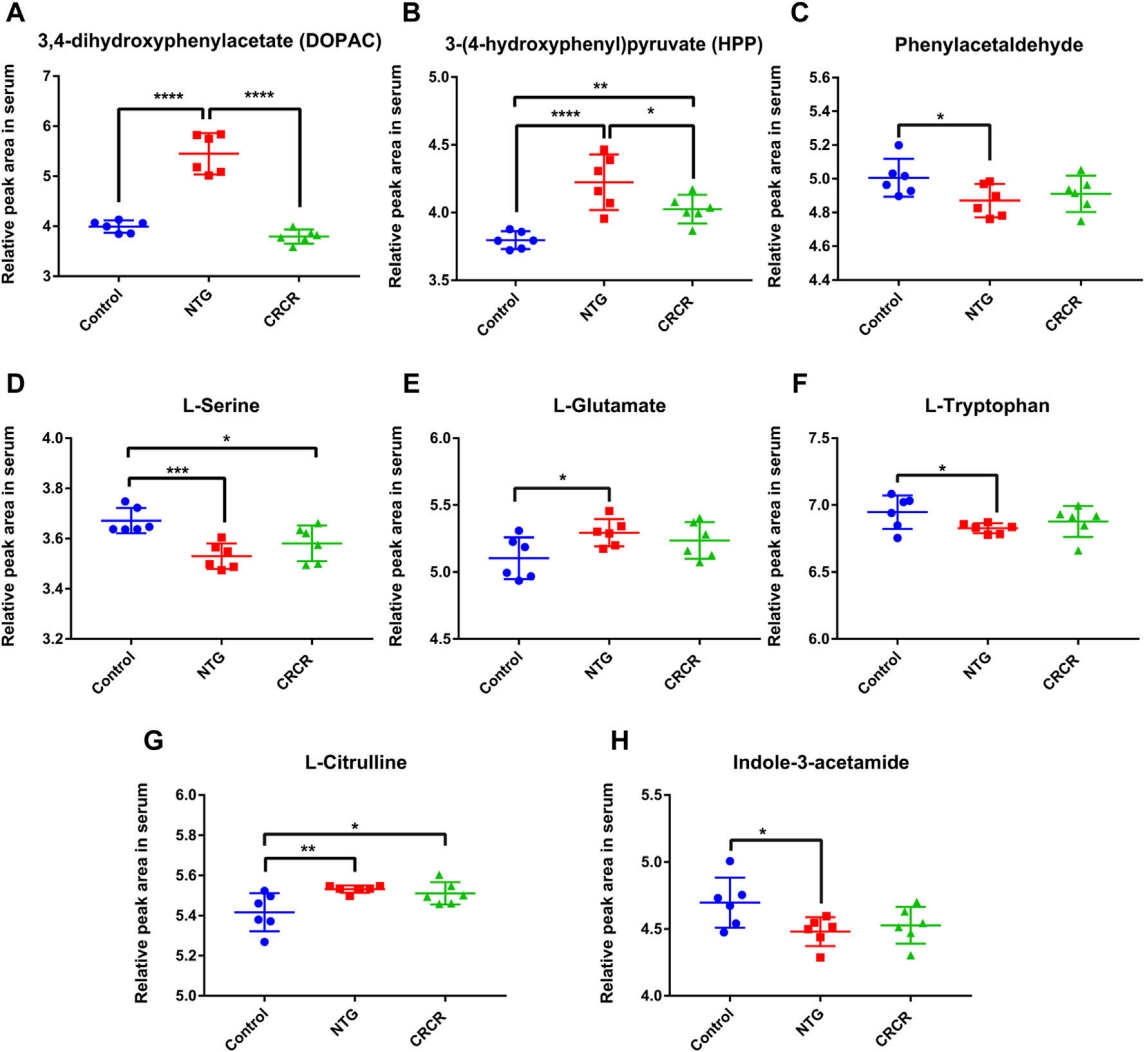
Comparison of relative peak areas of key differentially expressed metabolites in serum. **(A)** 3,4-Dihydroxyphenylacetate (DOPAC). **(B)** 3-(4-Hydroxyphenyl)pyruvate (HPP). **(C)** Phenylacetaldehyde. **(D)** L-serine. **(E)** L-glutamate. **(F)** L-tryptophan. **(G)** L-citrulline. **(H)** Indole-3-acetamide. Data are expressed as mean ± S.D. *p < 0.05, **p < 0.01, ***p < 0.001, ****p < 0.0001.

Figure 9 shows the relative peak areas of some key differentially expressed metabolites in rat serum. Compared with control rats, the contents of DOPAC, HPP, L-glutamate, and L-citrulline were significantly increased in the migraine rats (*p* < 0.0001, *p* < 0.0001, *p* < 0.05, *p* < 0.01), while the contents of phenylacetaldehyde, L-serine, L-tryptophan, and indole-3-acetamide were significantly decreased in the migraine rats (*p* < 0.05, *p* < 0.001, *p* < 0.05, *p* < 0.05). Treatment with CRCR caused significant decreases in the contents of DOPAC and HPP (*p* < 0.0001, *p* < 0.05). However, the contents of phenylacetaldehyde, L-serine, L-glutamate, L-tryptophan, L-citrulline, and indole-3-acetamide were not statistically different between the NTG group and CRCR group. Thus, it can be speculated that DOPAC and HPP were the key endogenous metabolites for CRCR treating migraine.

### Correlation Analysis

**FIGURE 10:**
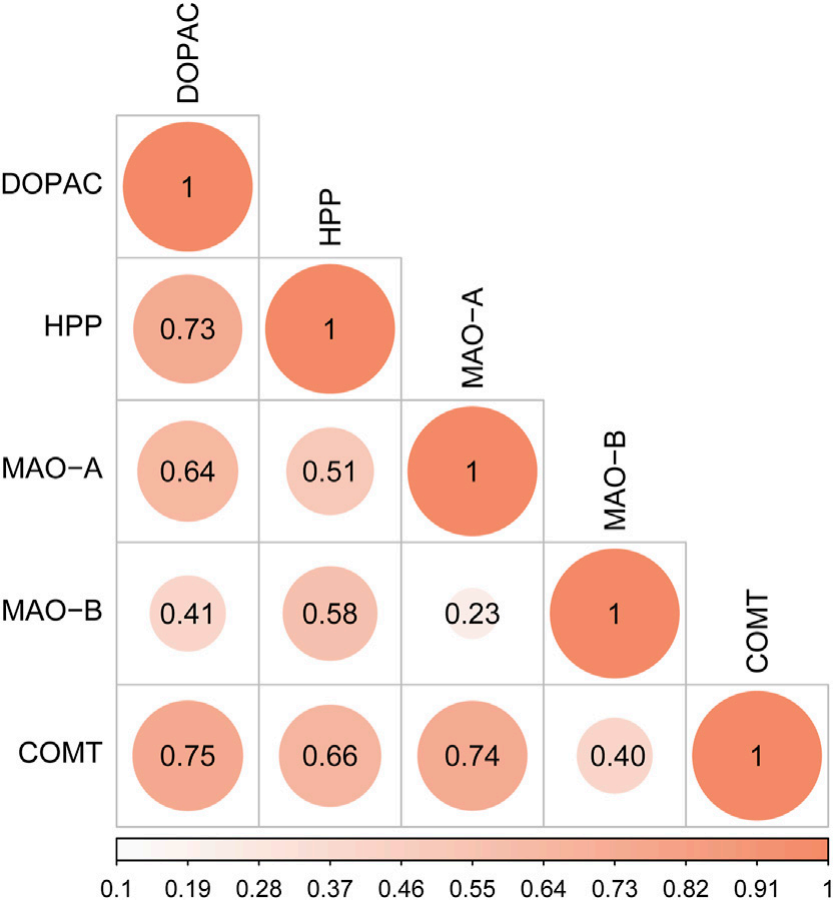
Correlation analysis of key differentially expressed metabolites (DOPAC and HPP) and key targets (MAO-A, MAO-B, and COMT).

Correlation analysis was conducted between the peak areas of key endogenous metabolites (DOPAC and HPP) and the contents of key targets (MAO-A, MAO-B, and COMT). The values of Pearson correlation coefficient are shown in Figure 10. DOPAC and HPP, the products of tyrosine metabolism, were closely related to each other. DOPAC and HPP had a higher correlation with COMT than MAO. These results confirmed that DOPAC and HPP were highly positively correlated with MAO-A, MAO-B, and COMT in the tyrosine metabolism pathway.

## Discussion

The pathological mechanisms of migraine are very complicated, which have been associated with neurogenic inflammation, central sensitization, trigeminovascular damage, oxidative stress, and metabolic abnormalities (Guo et al., 2017). Recent studies have suggested that the abnormalities of tyrosine metabolism are present in the plasma of episodic migraine without aura and cluster headache patients (high level of dopamine and low level of norepinephrine) (Akerman and Goadsby, 2007; Zarcone and Corbetta, 2017). Tryptamine level in plasma is significantly lower in migraineurs (D'Andrea et al., 2015). Glutamate is higher in the brain and possibly also in the peripheral circulation in migraine patients, particularly during attacks (Hoffmann and Charles, 2018). The levels of arginine, homoarginine, and citrulline were significantly lower in chronic cluster headache patients (D’Andrea et al., 2019). Accordingly, it is necessary to explore metabolic abnormalities in migraine attacks. NTG administration produces attacks similar to spontaneous migraine attacks and sensitizes trigeminal and cortical structures that underline migraine allodynia (Di Clemente et al., 2009). CRCR is a classic prescription for treating migraine, but its molecular targets are still no clear. In the present study, an NTG-induced migraine rat model was used to reveal the potential mechanisms of CRCR extract against migraine using integrated analysis of metabolomics and network pharmacology.

As reported, a large number of active components were found in CRCR, including phthalides, alkaloids, organic acids, terpenes, flavonoids, phenylpropanoids, and so on. These components showed various pharmacological activities. For example, ferulic acid has essential biological activities in oxidative stress, inflammation, apoptosis, and platelet aggregation (Nabavi et al., 2015; Li et al., 2021). Senkyunolide A, 3-n-butylphthalide, and ligustilide inhibit the production of proinflammatory mediators in lipopolysaccharide-stimulated murine BV-2 microglial cells and human peripheral blood monocyte-derived macrophages (Or et al., 2011; He Z. et al., 2017). Ligustilide alleviates brain damage and improves cognitive function in rats of chronic cerebral hypoperfusion (Feng et al., 2012). Butylidenephthalide improves motor symptoms and protects dopamine neurons in a mouse model of Parkinson’s disease (Chi et al., 2018). Nookatone improves cognitive impairment in Aβ _1-42_-induced Alzheimer’s disease model mice with inhibitory effect against Aβ accumulation (He B. et al., 2017; Wang Y. et al., 2018). α-Cyperone binds to tubulin and alters the dynamics of its polymerization for reduction of inflammation response in brain (Azimi et al., 2016). It can be seen that main components in CRCR could inhibit neuroinflammation. The present study showed that CRCR significantly increased 5-HT and decreased CGRP and NOS in serum and brainstem tissue of the migraine rats, indicating that CRCR had a good antineuroinflammatory effect on NTG-induced migraine rats.

Metabolomics, as one of the major “omics” technologies, provides a powerful tool in the diagnosis of diseases, discovery of potential biomarkers, and exploration of disease pathogenesis. Based on metabolomics analysis, 96 differentially expressed metabolites were identified. Five key metabolic pathways were found, including tyrosine metabolism, aminoacyl-tRNA biosynthesis, arginine biosynthesis, glycine, serine, and threonine metabolism and tryptophan metabolism. Seven differentially expressed metabolites were enriched in the above metabolic pathways. Therefore, special attention was paid to seven differentially expressed metabolites (DOPAC, HPP, L-serine, L-glutamate, L-tryptophan, L-citrulline, and indole-3-acetamide).

Accumulating evidence has demonstrated that network pharmacology is an attractive tool to predict the interactions between multiple components and multiple targets in the botanical drugs (Li and Zhang, 2013). In recent years, many researchers have successfully applied network pharmacology to discover the molecular mechanisms (Ma et al., 2019; Liu et al., 2020). Nonetheless, network pharmacology is limited by the integrity and reliability of the public databases (Zhou et al., 2020). At the same time, network pharmacology could predict the possibility of the compound–target combination and molecular action (Yuan et al., 2017), but it needs sufficient experiments to support the predictive results. Based on network pharmacology analysis, 31 active compounds were chosen in CRCR, and 201 targets were identified as the potential targets for CRCR against migraine. GO and KEGG enrichment analyses indicated that multiple targets and multiple pathways were closely involved in CRCR treating migraine, including neurotransmitter biosynthetic process, monoamine transport, serotonergic synapse, and dopaminergic synapse. It is noteworthy that dopaminergic synapse was involved in the synthesis and degradation of dopamine. Dopamine is synthesized from tyrosine via L-3,4-dihydroxyphenylalanine (L-DOPA) (Valjent et al., 2019). Dopamine can be converted to noradrenaline via the action of dopamine β-hydroxylase or catabolized by the joint action of the enzymes of COMT and MAO (Berke, 2018).

The differentially expressed metabolites found in metabolomics were further analyzed to obtain the corresponding targets by a compound–reaction–enzyme–gene network. By intersecting 77 targets in the compound–reaction–enzyme–gene network with 201 targets in the protein–protein interaction network, MAO-A, MAO-B, and COMT were identified as the common targets. Interestingly, MAO-A, MAO-B, and COMT are the essential targets involved in the tyrosine metabolism pathway (D’Andrea et al., 2015). A number of studies, conducted in the last decades, have demonstrated that a complex enzyme abnormality of tyrosine metabolism occurs in migraine patients with a possible derangement in the synthesis of neurotransmitters and neuromodulators (D’Andrea et al., 2007; D’Andrea and Leon, 2010). Tyrosine is the amino acid precursor for the synthesis of catecholamines, including 3,4-dihydroxyphenylalanine (DOPA), dopamine, norepinephrine, epinephrine, and elusive amines, such as tyramine, octopamine, and synephrine (D’Andrea et al., 2012). The abnormal levels of all the products of tyrosine metabolism may constitute the metabolic events that predispose to the occurrence of cluster headache and migraine attacks (D’Andrea et al., 2013). DOPAC and HPP, two key metabolites in the tyrosine metabolism, were significantly increased in the migraine rats, and CRCR caused significant decreases in the contents of DOPAC and HPP. MAO-A, MAO-B, and COMT are the key enzymes in the tyrosine metabolism. Thus, the contents of MAO-A, MAO-B, and COMT were further analyzed. Treatment with CRCR significantly decreased the levels of MAO-A and COMT in rat serum and brainstem tissue, while no significant difference was found in MAO-B. MAO is a mitochondrial-bound enzyme, which catalyzes the oxidative degradation of a series of monoamine neurotransmitters (Carradori et al., 2014). MAO-A and MAO-B exhibit different substrate specificities. Serotonin, dopamine, and norepinephrine are more efficiently oxidized by MAO-A, while phenethylamine, benzylamine, and octopamine are primarily metabolized by MAO-B (Edmondson and Binda, 2018). COMT enzyme is involved in the methylation of catechol substrates, classically in the metabolism of dopamine, norepinephrine, and epinephrine both in the periphery and the central nervous system (Witte and Flöel, 2012). Maybe due to the specific selection in substrates, the contents of MAO-A and COMT changed significantly with the treatment of CRCR, but MAO-B was not.

In the present study, DOPAC and HPP, the key metabolites involved in the tyrosine metabolism, were significantly increased in the migraine rats. MAO-A and COMT, the key enzymes involved in the tyrosine metabolism, were also significantly increased in the migraine rats. CRCR extract sharply reduced the contents of DOPAC and HPP, as well as MAO-A and COMT. Correlation analysis showed that DOPAC and HPP were highly positively correlated with MAO-A and COMT in the tyrosine metabolism pathway. Accordingly, it can be supposed that two key differentially expressed metabolites (DOPAC and HPP), two key targets (MAO-A and COMT), and one key metabolic pathway (tyrosine metabolism) were closely involved in the treatment of migraine by CRCR extract.

## Conclusion

The present study explored the potential mechanisms of CRCR for migraine treatment using integrated analysis of metabolomics and network pharmacology. The integrated analysis revealed that two key differentially expressed metabolites (DOPAC and HPP), two key targets (MAO-A and COMT), and one relevant metabolic pathway (tyrosine metabolism) showed great importance in CRCR treating migraine. This research could provide a new understanding of the potential mechanism of CRCR against migraine. More attention should be paid into the tyrosine metabolism pathway in future studies.

## Data Availability

The original contributions presented in the study are included in the article/Supplementary Material, further inquiries can be directed to the corresponding author.

## Supplementary Material

Supplementary Figure S1 The total ion chromatograms of the quality control (QC) samples in the positive **(A)** and negative ion modes **(B)**.

## Abbreviations

CGRP, calcitonin gene-related peptide; COMT, catechol-O-methyltransferase; CRCR, Conioselinum anthriscoides “Chuanxiong” (Apiaceae; Chuanxiong rhizoma) and *Cyperus* rotundus L. (Cyperaceae; Cyperi rhizome); DOPA, 3,4-dihydroxyphenylalanine; DOPAC, 3,4-dihydroxyphenylacetate; HPP, 3-(4-hydroxyphenyl)pyruvate; MAO-A, monoamine oxidase A; MAO-B, monoamine oxidase B; NO, nitric oxide; NOS, nitric oxide synthase; NTG, nitroglycerin; OPLS-DA, orthogonal partial least squares discriminate analysis; PLS-DA, partial least squares discriminate analysis; QC, quality control; UHPLC-Q-extractive MS, ultrahigh-performance liquid chromatography Q Exactive mass spectrometry; 5-HT, 5-hydroxytryptamine.
