# Supplementary material for: Reveal the Antimigraine Mechanism of Chuanxiong Rhizoma and Cyperi Rhizoma Based on the Integrated Analysis of Metabolomics and Network Pharmacology
Source: Front Pharmacol. 2022 Mar 24;13:805984. doi: 10.3389/fphar.2022.805984 (PMC8987590; doi:10.3389/fphar.2022.805984)
Supplement: Supplementary file 1 [file Table1.pdf]

Table S1. 77 potential target genes obtained from the compound-reaction-enzyme-gene network.

Gene Symbol										
AASDHPPT	ALDH3B1	ASNS	CRAT	FPGS	GGT1	GLS2	GPT	NAGS	PSPH	SPTLC2
AGXT	ALDH3B2	ASS1	DDAH1	FTCD	GGT2	GLUD1	GPT2	OPLAH	SARS	TAT
AGXT2	ALDH4A1	AZIN2	DDAH2	GAD1	GGT3	GLUD2	HPD	OTC	SARS2	TDO2
ALDH18A1	ALDH8A1	BAAT	DDC	GAD2	GGTL3	GLUDP5	IL4I1	PEPB	SDS	TPH1
ALDH1A1	AOC1	BTD	EARS2	GATM	GGTL4	GLUL	MAOA	PLD1	SHMT1	TPH2
ALDH1A3	AOC2	CBS	EPRS	GCLC	GGTLA1	GOT1	MAOB	PLD2	SHMT2	WARS
ALDH3A1	AOC3	COMT	FAAH2	GCLM	GLS	GOT2	MIF	PRKACA	SPTLC1	WARS2
