# Supplementary figures and images for: Reveal the Antimigraine Mechanism of Chuanxiong Rhizoma and Cyperi Rhizoma Based on the Integrated Analysis of Metabolomics and Network Pharmacology
Source: Front Pharmacol. 2022 Mar 24;13:805984. doi: 10.3389/fphar.2022.805984 (PMC8987590; doi:10.3389/fphar.2022.805984)

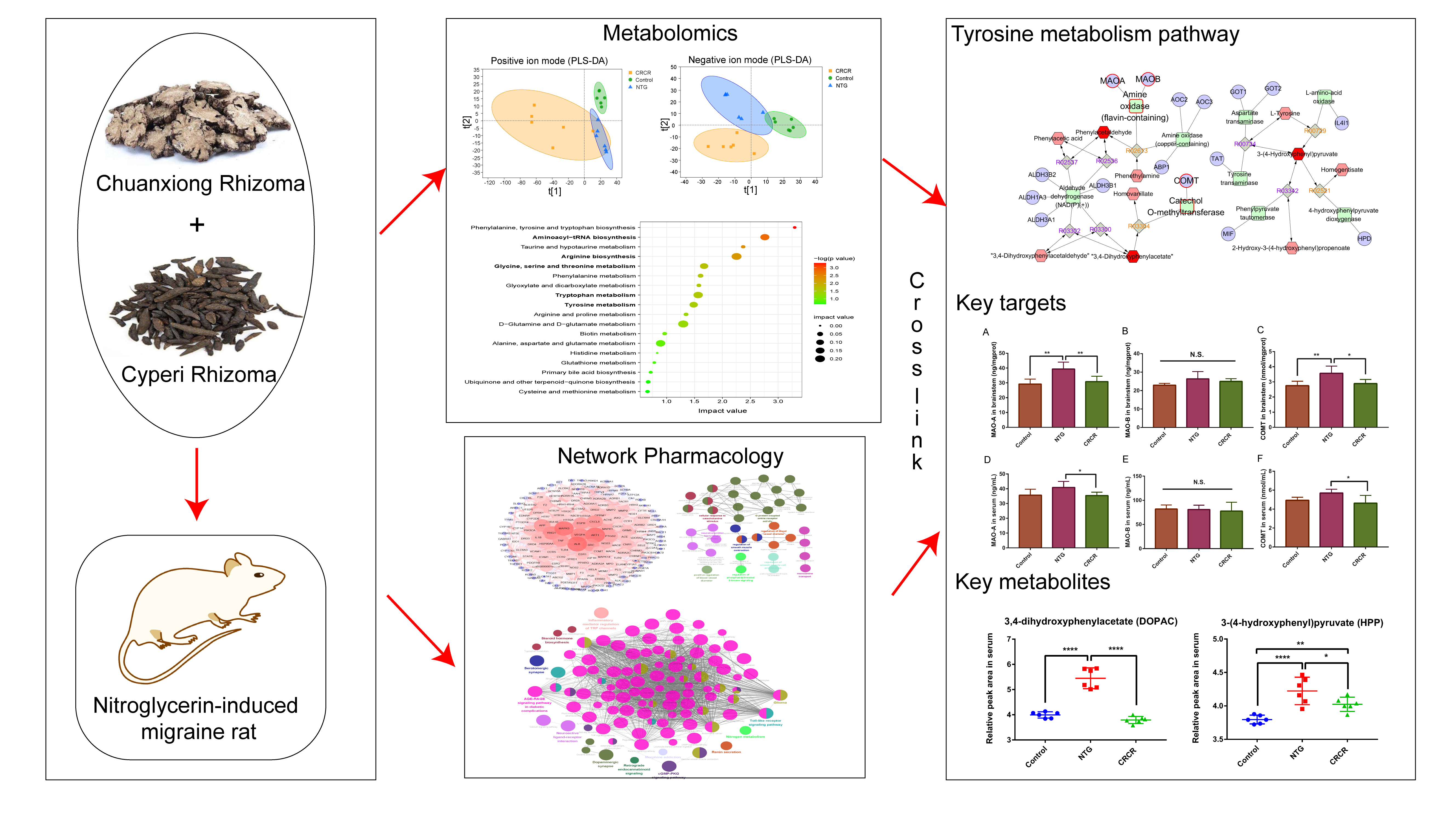

Supplement: Supplementary file 2 [file Image2.TIF]

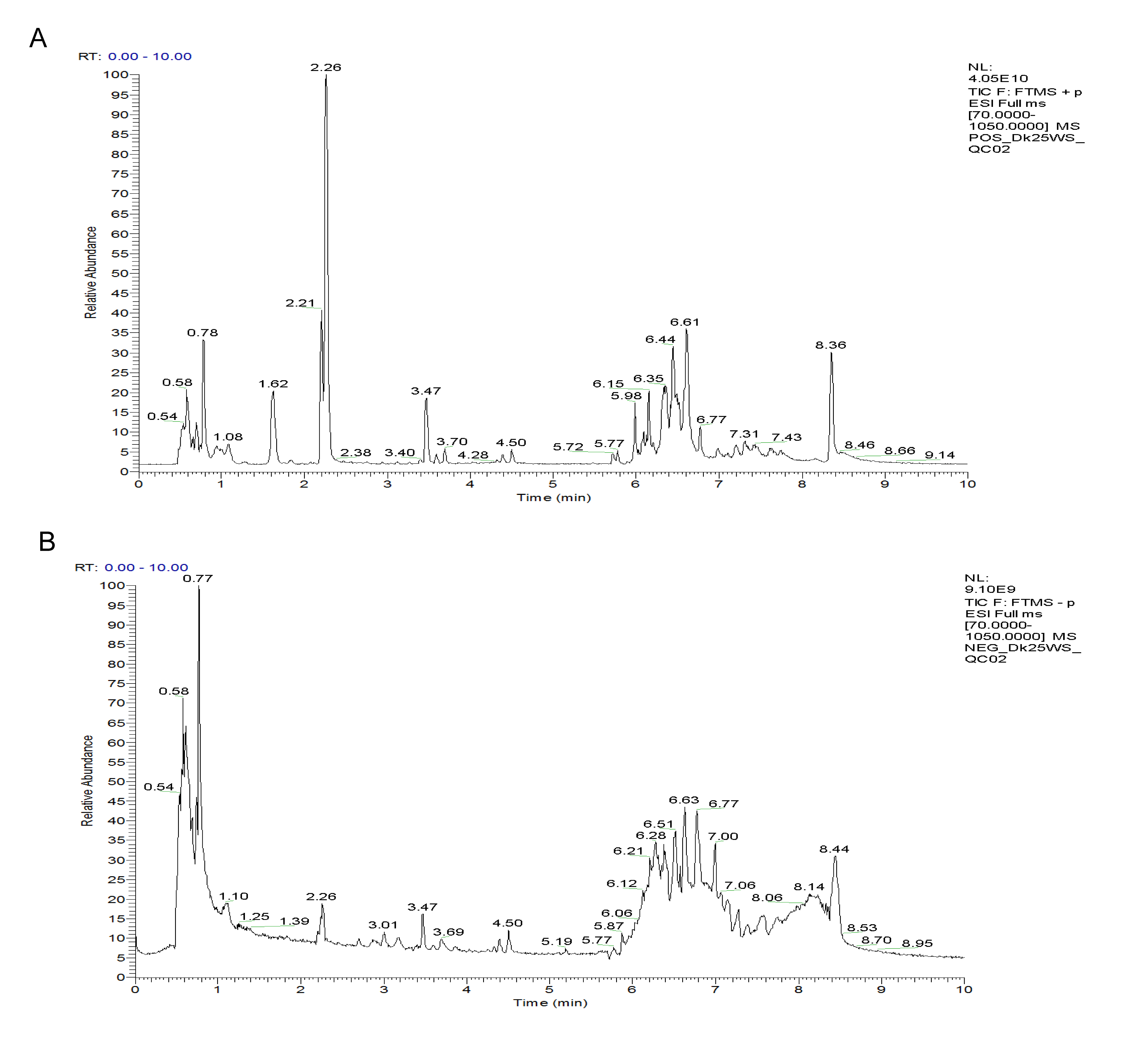

Supplement: Supplementary file 3 [file Image1.TIF]
